# Individual differences and creative ideation: neuromodulatory signatures of mindset and response inhibition

**DOI:** 10.3389/fnins.2023.1238165

**Published:** 2023-12-06

**Authors:** Radwa Khalil, Sergio Agnoli, Serena Mastria, Angela Kondinska, Ahmed A. Karim, Ben Godde

**Affiliations:** ^1^School of Business, Social and Decision Sciences, Constructor University, Bremen, Germany; ^2^Department of Life Sciences, University of Trieste, Trieste, Italy; ^3^Marconi Institute for Creativity, Sasso Marconi, Italy; ^4^Department of Psychology, University of Bologna, Bologna, Italy; ^5^Department of Psychiatry and Psychotherapy, University Clinic Tübingen, Tübingen, Germany; ^6^Department of Health Psychology and Neurorehabilitation, SRH Mobile University, Riedlingen, Germany

**Keywords:** individual differences, mindset, tDCS, creative ideation, response inhibition, neuromodulation, divergent thinking

## Abstract

This study addresses the modulatory role of individual mindset in explaining the relationship between response inhibition (RI) and divergent thinking (DT) using transcranial direct current stimulation (tDCS). Forty undergraduate students (22 male and 18 female), aged between 18 and 23 years (average age = 19 years, SD = 1.48), were recruited. Participants received either anodal tDCS of the right IFG coupled with cathodal tDCS of the left IFG (R + L−; *N* = 19) or the opposite coupling (R−L+; *N* = 21). We tested DT performance using the alternative uses task (AUT), measuring participants’ fluency, originality, and flexibility in the response production, as well as participants’ mindsets. Furthermore, we applied a go-no-go task to examine the role of RI before and after stimulating the inferior frontal gyrus (IFG) using tDCS. The results showed that the mindset levels acted as moderators on stimulation conditions and enhanced RI on AUT fluency and flexibility but not originality. Intriguingly, growth mindsets have opposite moderating effects on the change in DT, resulting from the tDCS stimulation of the left and the right IFG, with reduced fluency but enhanced flexibility. Our findings imply that understanding neural modulatory signatures of ideational processes with tDCS strongly benefits from evaluating cognitive status and control functions.

## Introduction

### Creative ideation and response inhibition

Several candidates have been put forward to explain the information-processing mechanisms that underlie individual differences in creativity (Schneider and Shiffrin, 1977; Vartanian et al., 2007; Dorfman et al., 2008; Colom et al., 2010; Abraham, 2018). One of the potential explanatory mechanisms at the basis of individual differences in creativity is the *disinhibition hypothesis* (Hodin, 1954); highly creative individuals utilize cognitive disinhibition to access ordinarily hidden associations from conscious awareness (Schnier and Kris, 1953; Koestler, 1975; Eysenck, 1995; Carson, 2014). Studies suggest that high cognitive control supports the selection and actuation of more effective task strategies and suppresses interference from common and inappropriate response tendencies (Beaty and Silvia, 2012), which supports specific creative performances (Benedek and Fink, 2019). In terms of inhibiting salient but irrelevant responses, cognitive control appears to facilitate creative ideation. Inhibitory control (IC)^1^ is one of the core elements characterizing cognitive control in creative ideation (Benedek et al., 2012; Zabelina et al., 2019; Khalil et al., 2020, 2023a; Palmiero et al., 2022). It refers to suppressing increasing proactive interference of previous responses and resisting interference from potentially attention-capturing processes or contents (Harnishfeger, 1995; Diamond, 2013) to promote steady access to unrelated concepts and ideas, allowing for higher creativity.

There is a common agreement that IC involves cognitive and behavioral inhibition (Aron, 2007). Cognitive inhibition suppresses previously activated cognitive contents or processes, removes irrelevant acts or attention from consciousness, and resists attention-capturing processes or contents (Harnishfeger, 1995). Therefore, cognitive inhibition refers to inhibiting thoughts and memories as interference control (Diamond, 2013).

Behavioral inhibition includes (a) selective or focused attention, which refers to inhibition at the level of attention and hence attentional inhibition, and (b) response inhibition (RI), which refers to inhibition at the level of behavioral responses or motor actions and thus, to self-control and discipline (Diamond, 2013). At the neural level, it has been suggested that inhibitory processes fall within fronto-striatal neural circuits, including the basal ganglia and dorsolateral and ventrolateral parts of the prefrontal cortex (PFC; Aron, 2007; Munakata et al., 2012a,b; Bari and Robbins, 2013; Aron et al., 2014). People should immediately suppress spontaneous answers and explore unique thoughts to generate original ideas (Cassotti et al., 2016; Agnoli et al., 2020; Khalil et al., 2020). IC is, thus, a critical process for generating creative ideas. However, more research is required to fully comprehend the mechanistic relationship (Palmiero et al., 2022).

Recently, researchers using the transcranial direct current stimulation (tDCS) method highlighted particular brain dynamics sustaining IC that emerge to benefit creative thinking (Khalil et al., 2020; Li et al., 2022). Brain stimulation/neuromodulation techniques, such as tDCS, are gradually gaining recognition in the neuroscience of creativity (Lucchiari et al., 2018). These techniques facilitate the measurement of causality to understand the involvement of brain dynamics in the expression of creative behavior.

### Hemispheric modulation of divergent thinking by tDCS

Research has mostly examined the functional lateralization of creativity, and the validity of the *right brain hypothesis* for the neuroscience of creativity is a virtual battleground for creativity researchers (Dietrich, 2004, 2019; Zaidel, 2013; Abraham, 2018). Indeed, *the right brain hypothesis* has been questioned (Abraham et al., 2012; Aziz-Zadeh et al., 2013; Beaty et al., 2014, 2017, 2018; Abraham, 2018), as creativity is based on information processing across several brain networks (Beaty et al., 2015, 2016, 2019; Beaty and Schacter, 2017). The temporal dynamics of divergent thinking (DT) were associated with brain activity (especially in the alpha frequency band) in both the right and left posterior cerebral regions when cerebral dynamics predicted the emergence of several indices of DT, such as originality and flexibility (Agnoli et al., 2020; Mastria et al., 2022).

The persistence of the *creative right brain* idea is remarkable, especially considering the earliest advocates of lateralization research have highlighted the significance of the specificity of each hemisphere in creativity (Bogen and Bogen, 1969; Miran and Miran, 1984; Hoppe, 1988). Stimulation techniques can be used to manipulate brain activity in the two hemispheres, and several researchers have suggested that tDCS may have the potential to boost creativity, even if other studies did not show any effect of tDCS on creative performance (Lucchiari et al., 2018; see Table 1 of Li et al., 2022).

Research suggests that bilateral stimulation of the PFC has a greater influence on creative cognition than unilateral stimulation (Mayseless and Shamay-Tsoory, 2015; Khalil et al., 2020; PeÑa et al., 2021; Xiang et al., 2021). The study by Mayseless and Shamay-Tsoory (2015) indeed supported the *balance hypothesis,* which states that DT requires a balance of frontal lobe function (right and left IFG). Results of this study revealed that left cathodal (i.e., inhibitory) and right anodal (i.e., excitatory) stimulation enhanced DT scores, while the reverse condition did not.

It has been observed that right anodal and left cathodal stimulation (R + L−) improved fluency in the IFG (Mayseless and Shamay-Tsoory, 2015; Hertenstein et al., 2019; Khalil et al., 2020). IFG is the brain area involved in controlled retrieval from semantic memory (Binder et al., 2009), associative thinking (e.g., metaphor; Mashal et al., 2007), and creative writing (Shah et al., 2013). Other findings indicated that anodal stimulation over the dorsolateral PFC is more likely to contribute to originality relative to cathodal stimulation over the left ventrolateral PFC/inferior frontal gyrus (IFG) (Groborz and Necka, 2003; Beaty et al., 2016; Murray, 2017; Ghanavati et al., 2019; Huang et al., 2022). Stimulation site/protocol and target brain area variance may explain this inconsistency. Nevertheless, they suggested the complicated influence of both the left and right hemispheres on creative thinking.

### The role of individual differences in terms of mindset

To understand the individual differences in creative performance, we suggest a differential approach taking into account both cerebral dynamics and behavioral-attitudinal dynamics. In the present study, we focused specifically on the differential role exerted by individual mindsets on the DT brain dynamics as stimulated through tDCS. Our mindsets influence indeed how we perceive the world and ourselves (Dweck, 2006, 2012; Bittner and Heidemeier, 2013; Gilead et al., 2014). In her seminal work, Dweck (2006) contrasted a growth mentality with a fixed mindset. In fixed mindsets, people believe they cannot enhance their intellectual abilities and feel helpless when assigned complex tasks. Therefore, they refrain from trying new ideas or problem-solving methods. In contrast, a growth mindset promotes self-esteem, openness, and cognitive flexibility. Thus, a growth mindset may encourage creativity, unlike a fixed attitude. Changing one’s perspective from fixed to growing can readily increase creativity. As a result, evaluating the role of mindsets in the context of individual differences in creative-divergent performance should be taken into account. Although mindset is central to most decision-making and perception research (Schwabe, 2004; Bhanji and Beer, 2012; Gilead et al., 2014; Hege et al., 2018; Wang et al., 2018; Huijsmans et al., 2019; Muenks et al., 2020; Yu et al., 2020; Frank-Podlech et al., 2021; Hecht et al., 2021), it is still relatively ignored in the creativity literature.

### Study hypotheses

The present work started from the results obtained in a previous study (Khalil et al., 2020), which revealed that IFG brain activity was altered by tDCS as an explanatory neural mechanism of the link between RI and DT. In the current work, we address a different research question using the data collected in the previous research, considering mindset as a prospective moderator. Adding to the previous research (Khalil et al., 2020), we implemented additional scoring methods for DT performance in a more comprehensive modality. DT indeed quantifies participants’ ideational indices, such as fluency (numbers of produced ideas), flexibility (numbers of semantic categories used to produce alternative ideas), and originality—a reliable measure of creative potential (Guilford, 1950; Runco and Acar, 2012; see also Agnoli et al., 2023).

In the research by Khalil et al. (2020), RI predicted DT performance by modulating left-to-right IFG activity efficiency; nevertheless, tDCS stimulation did not improve all creative ideation scores. For example, increased originality and flexibility, but not fluency, resulted from cathodal-tDCS (c-tDCS) stimulation targeting the left IFG coupled with anodal-tDCS (a-tDCS) over the right IFG (R + L−). Remarkably, RI had a moderation effect, as tDCS only enhanced originality and flexibility in the stimulation condition of R + L− when d’ (a measure extracted from the Go No Go Task-GNGT- measuring RI) was enhanced. This finding initiated a follow-up question of whether this enhanced d’ level (i.e., the moderation effect of RI) might reflect a specific state of mind that facilitates or attenuates the effects of tDCS on DT. The dependencies of the tDCS effect on the neuro-cognitive states of the participants have been suggested previously (Learmonth et al., 2015; Hsu et al., 2016). We expect that individual differences in mindset, as a cognitive state of mind, would help explain part of the variance of the moderation effect of d′ on the changes induced by tDCS on DT.

For this reason, we hypothesized that the participants’ mindset level could moderate the effect of tDCS and RI on the creative performance indices. Specifically, we expected that the change in creative performance induced by tDCS stimulation would not be influenced only by the RI level, as demonstrated in the previous study (Khalil et al., 2020), but also by participants’ mindsets. Whereas we expected to find the main and interactive effects of tDCS and RI on the change in DT, as an exploratory hypothesis - due to the lack of previous literature on the topic - we also expected an interaction effect of mindset with the previous variables on DT performance.

## Methods

### Participants

The sample included 40 undergraduate students (22 males and 18 females) from Constructor University (formerly Jacobs University) who were compensated with course credits or monetary rewards. Age ranged from 18 to 23 years (M = 19, SD = 1.48). The experimental protocol conformed to the Declaration of Helsinki and followed local ethical standards and German law. All participants underwent an eligibility screening for the tDCS, had no previous experience with this experiment’s materials and tDCS procedures, and gave written informed consent before starting the experiment. Participants with neurologic, medical, or psychiatric disorders were excluded from the study. All participants were right-handed based on the Edinburgh inventory assessments (Oldfield, 1971).

### Mindset questionnaire

Based on Dweck (2006) growth mindset research, the Mindset Questionnaire was adapted from Diehl (2008) study (Appendix). This questionnaire was retrieved from http://www.classroom20.com/forum/topics/motivating-students-with.

This self-reported questionnaire contained 20 items assessing participants’ mindsets; a four-point Likert scale; participants had to choose “strongly agree,” “agree,” “disagree,” or “strongly disagree.” After evaluating the four Likert scale questionnaires, the participants’ scores ranged from 20 to 49 points.

### Alternative uses task

The paper and pencil version of the Alternative Uses Task (AUT; Guilford et al., 1960) was used. Each participant was asked to write down alternative uses for two everyday objects (i.e., brick and paperclip) within 2 min (*cf.* Mayseless and Shamay-Tsoory, 2015) after becoming familiar with an example of the uses for a newspaper. The words of the objects were all new in the pre-and post-tests and counterbalanced across participants.

Three measures of participants’ creative performance were derived from the AUT: fluency, flexibility, and originality (using external raters). Fluency was scored as the total number of valid responses generated by each participant. Flexibility was calculated by averaging the total number of conceptual categories utilized per object at the subject level according to pre-existing categories extracted *ad hoc* based on our data set (Reiter-Palmon et al., 2019), weighted for each participant’s total number of responses. Originality was calculated by involving two raters who independently judged the originality of each participant’s response (Silvia et al., 2008), showing good consistency and agreement with each other (Inter-rater reliability was operationalized via intraclass correlations (ICCs) across the two raters). ICCs ranged from 69 to 1.00 for AUT measures (fluency, flexibility, and originality). For each object, the responses were transcribed into a spreadsheet and alphabetically ordered within each object. The ID associated with each participant was then hidden. This procedure guaranteed that the serial position of the ideas did not bias the evaluation, as did the total number of the ideas in the set, the participant who generated the idea, and the preceding and successive ideas. The judges were asked to read all the responses before scoring them. Response originality was rated on a 1 (= not at all original) to 5 (= highly original) scale, according to one of the most accepted scoring methods (Silvia et al., 2008). Raters were instructed to consider and weigh three dimensions, i.e., uncommonness, remoteness, and cleverness (Forthmann et al., 2017). Specifically, uncommonness refers to the fact that an idea is not ordinarily encountered in participants’ responses; remoteness refers to the distance of an idea from what is commonly thought; and cleverness includes the concepts of imaginativeness, smartness, and funniness (Silvia et al., 2008; see also Forthmann et al., 2017). Only when ideas were evaluated highly on all three dimensions did raters score them as highly original (i.e., 5). In cases of enormous discrepancies between ratings, the judges reviewed their responses and assigned a score by consensus. The mean originality score for each idea was calculated from the ratings of the two raters. An average originality score per participant was obtained as the average originality for the two objects.

### Go-NOGO task

The GNGT was implemented as described in the study by Swick et al. (2008). In Times New Roman size 140 font, lowercase black letters were presented on a white screen. The letter x represented the NoGo stimulus; in contrast, all the other alphabet letters referred to the Go stimuli. The participants were informed about pressing a lever once the Go stimulus was presented while inhibiting their response to the NoGo stimulus. The duration of stimuli was 200 ms, and interstimulus intervals were 1,500 ms. Two paradigms were used based on the proportion of Go/No-Go trials (50/50 and 90/10). The 50/50 paradigm included 70 Go/70 NoGo, while the 90/10 paradigm involved 126 Go/14 NoGo. The participants were familiarized with GNGT through a short practice set of 30 trials (50/50 Go/NoGo stimuli, randomly intermixed) and had a minute break between the 2 blocks.

Based on signal detection theory (Green and Swets, 1966), we used hit rate (HR) and false alarm (FA) rate to calculate the sensitivity index d-prime (d′) for accuracy, where increasing values of d′ refer to higher sensitivity to a given signal (i.e., GO stimuli). Using the linear log approach of Hautus (1995), HR and FA were adjusted to avoid values of 0 and 1, whereby 0.5 was added to HR and FA and 1 was added to Go and NoGo trials. The difference between the standardized (Z-transformed) HR and FA rate probability (d′ = zHR – zFA; Stanislaw and Todorov, 1999) is computed as d’.

### Transcranial direct current stimulation

**Figure 1:**
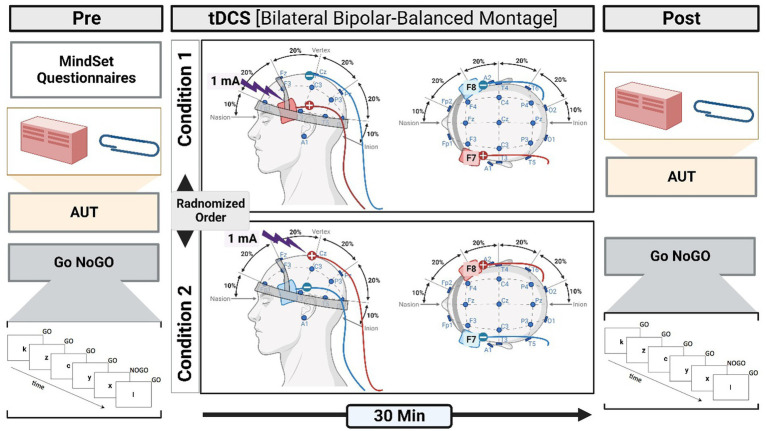
A schematic illustration of the methodological procedure. The experiment is designed to determine the effect of tDCS (pre- and post-) on DT through measuring mindset and RI. Pre-tDCS measures involve the mindset questionnaire, AUT, and Go NoGO (GNGT). The participants were randomly assigned to two conditions. In one condition, a-tDCS was applied to the right IFG and c-tDCS to the left IFG. In the second condition, the other half of the participants received the opposite coupling. After the tDCS (post-tDCS), the performance in AUT and GNGT was measured.

A battery-driven stimulator (Schneider Electronic, Gleichen, Germany) was used to apply tDCS over the left and right IFG, as shown in Figure 1. A constant current of 1 mA was applied via two saline-soaked sponge electrodes covering an area of 4 × 6 cm. tDCS was applied for 30 min with a 10 s ramp up and down (Nitsche and Paulus, 2000; Nitsche et al., 2003).

An EEG cap was used to localize and mark the positions of F7 and F8 on the scalp, which were associated with the left and right IFG, respectively (Ozawa et al., 2014). We chose a bilateral bipolar-balanced montage to stimulate the left IFG (F7) and inhibit its right-hemispheric counterpart (F8) and vice versa (Neuling et al., 2012; Nasseri et al., 2015). This bilateral configuration resembles that used in previous studies on creativity (Chrysikou et al., 2013; Mayseless and Shamay-Tsoory, 2015; Lucchiari et al., 2018; Khalil et al., 2020) and is particularly suitable to test *the balance hypothesis* (Mayseless and Shamay-Tsoory, 2015; Khalil et al., 2020, 2023b).

### Procedure

Once participants had read the participant information sheet and signed the consent form, they were asked to complete a mindset self-report questionnaire. All participants took part in two tasks, namely, AUT and GNGT, before and after brain stimulation by tDCS. Participants were randomly divided into two stimulation groups (which we referred to as two conditions): one condition refers to anodal stimulation of right IFG coupled with cathodal stimulation of left IFG (i.e., R + L−), and the second condition refers to cathodal stimulation of right IFG coupled with anodal stimulation of left IFG (i.e., R−L+); Figure 1.

After the experiment, participants were orally examined to ensure they could not distinguish between the stimulation conditions. Potential tDCS side effects were evaluated with a questionnaire administered immediately at the end of the experimental session. All participants were verbally debriefed after the experimental session. The duration of the experiment was around 60 min, excluding 5 min for the introduction, the overview of the experiment, and signing the consent form.

### Data analysis

To investigate the influence of the direction of tDCS stimulation (left vs. right IFG), the RI level, participants’ mindset, as well as their interactions, on the change in the creative-divergent performance, changes in originality, fluency, and flexibility were evaluated in three separate generalized linear mixed (GLM) models and treated as dependent variables using SPSS software, version 26. Robust error estimation was used to rule for the possible effect of outliers and violations of normality assumptions (Wu, 2009), controlling for the random effect of subjects.

Given that we were interested in the change in the creative performance between the two measurement sessions before and after the stimulation, an index of the change for each creative measure was derived by computing the difference (delta ∆) between the pre-stimulation performance and the post-stimulation performance in DT: positive values indicate an increase in creative performance after the stimulation, whereas negative values indicate a decrease in performance.

A series of GLM models (one per each creative performance index: ∆originality, ∆flexibility, and ∆fluency) were performed, entering tDCS CONDITION (two levels: R + L−, R−L+) as between-subjects fixed factor. The interactions (two and three-way) between tDCS CONDITION, RESPONSE INHIBITION (as measured through d’delta 90 as derived from the GNGT in the pre-stimulation session), and MINDSET (as measured through the scores obtained in the 4-point Likert scale) were added as fixed interaction factors to the models. Data and analysis code for this study are available upon request to the corresponding author.

## Results

### Originality

The first GLM model on the changes in originality did not show any significant main or interaction effects (all F*s* < 1.748, *ps* > 0.191; see Supplementary Table).

### Flexibility

**Figure 2:**
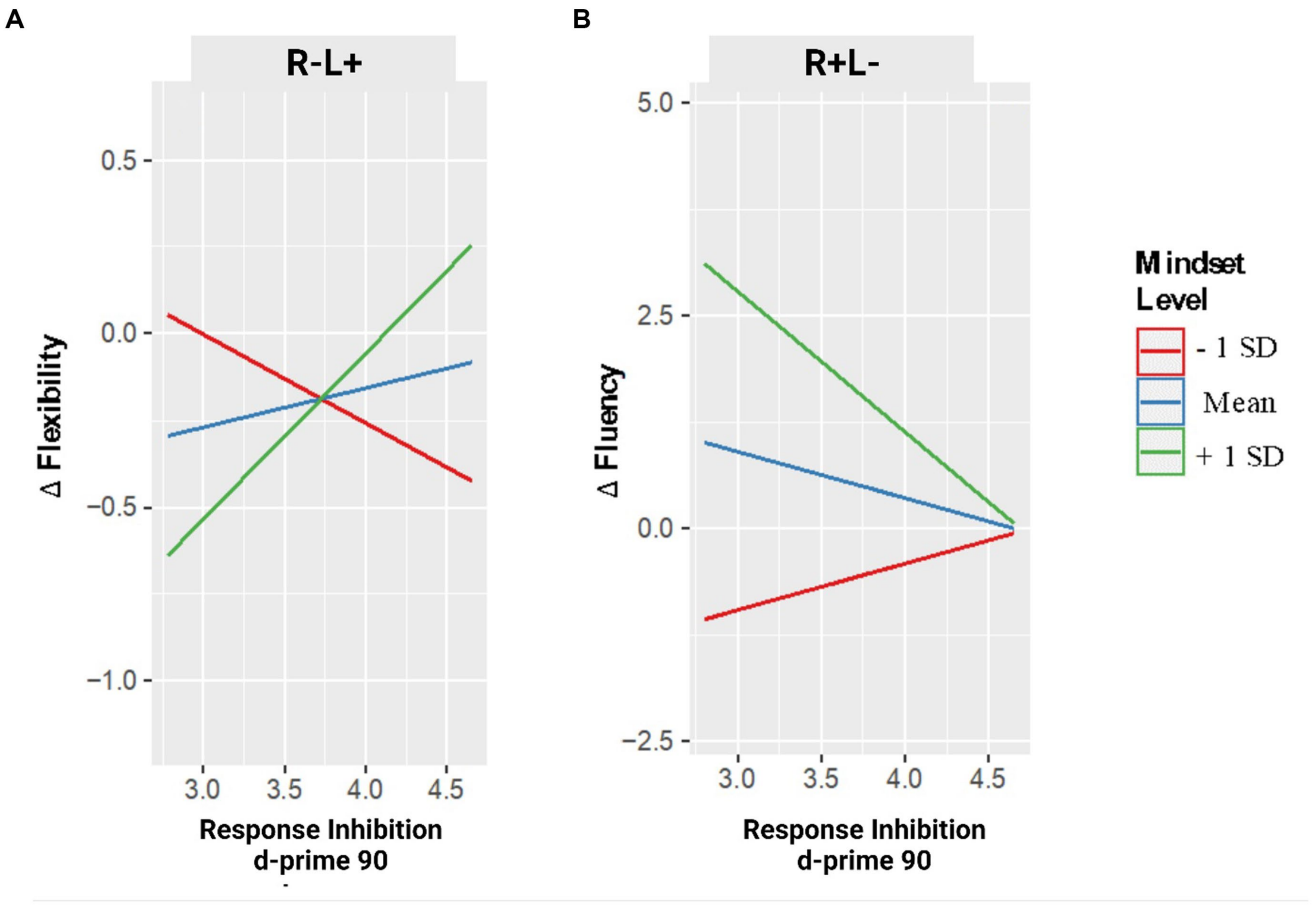
Changes in creative performance (flexibility – panel **A** – and fluency – panel **B**) as a function of RI are measured in GNGT and the Mindset level. Note. Changes in flexibility and fluency are expressed in delta values: positive values indicate an increase in performance after the stimulation compared to the pre-stimulation condition; negative values indicate a decrease in performance after the stimulation.

The GLM model on flexibility first showed a main effect of the tDCS CONDITION, revealing that the stimulation of the left IFG was associated with an increase of ideational flexibility, *F*_1,31_ = 5.617, *p* = 0.024, *b* = 5.571, 95% CI = [0.777, 10.364]; Figure 2A.

Moreover, two-way interaction between tDCS CONDITION and MINDSET emerged (*F*_2,31_ = 3.945, *p* = 0.03), which showed that in the tDCS stimulation of the left IFG (R−L+ condition), an increase of mindset scores (i.e., from fixed to growth) was associated with a decrease of flexibility, *b* = −0.158, *t*_2.31_ = −2.780, *p* = 0.009, 95% CI = [−0.274, −0.042], whereas no effect of mindset emerged in the right (R + L−) IFG stimulation condition, *b* = 0.016, *t*_2.31_ = 0.404, *p* = 0.689, 95% CI = [−0.066, 0.099].

This model showed a two-way interaction between tDCS CONDITION and RESPONSE INHIBITION (*F*_2,31_ = 3.391, *p* = 0.03), which highlighted that with the tDCS stimulation of the left IFG, an increase of RI was associated with a decrease in flexibility, *b* = −1.390, *t*_2.31_ = −2.757, *p* = 0.010, 95% CI = [−2.418, −0.362], whereas no effect of RI level emerged in the tDCS stimulation of the right IFG, *b* = 0.180, *t*_2.31_ = 0.550, *p* = 0.586, 95% CI = [−0.486, 0.846]. Nevertheless, these two-way interactions were further explained by a three-way interaction between tDCS CONDITION, MINDSET, and RESPONSE INHIBITION, *F*_2,31_ = 4.052, *p* = 0.027. Specifically, whereas with the right IFG stimulation, no interaction between MINDSET and RESPONSE INHIBITION emerged (*p* = 0.607), in the left IFG stimulation condition, analyses revealed an interaction between MINDSET and RESPONSE INHIBITION, *b* = 0.042, *t*_2.31_ = 2.799, *p* = 0.009, 95% CI = [0.011, 0.073].

As shown in Figure 2A, a simple slopes analysis emphasized the moderator role of participants’ mindset on RI over its association with flexibility, *b* = 0.043, *SE* = 0.018, *t* = 2.301, *p* = 0.036; specifically, after the stimulation of the left IFG, an increase of RI was associated with an increase of flexibility only at high mindset scores (i.e., growth mindset), *b* = 0.477, *SE* = 0.205, *t* = 2.327, *p* = 0.034. In contrast, no association between RI and flexibility emerged at medium (i.e., neither fixed nor growth mindset), *b* = 0.112, *SE* = 0.167, *t* = 0.671, *p* = 0.512, and at low (i.e., growth mindset) mindset scores, *b* = −0.253, *SE* = 0.253, *t* = −1.0092, *p* = 0.332.

### Fluency

The final GLM model on the changes in fluency showed a two-way interaction between tDCS CONDITION and MINDSET (*F*_2,31_ = 6.950, *p* < 0.01). In the tDCS stimulation of the right IFG (R + L− condition) an increase of mindset scores (i.e., from fixed to growth) was associated with an increase in fluency, *b* = 0.584, *t*_2.31_ = 3.700, *p* = 0.001, 95% CI = [0.262, 0.906], whereas no effect of mindset emerged in the left (R−L+) IFG stimulation condition, *b* = 0.136, *t*_2.31_ = 0.456, *p* = 0.650, 95% CI = [−0.486, 0.740].

This model showed a two-way interaction between tDCS CONDITION and RESPONSE INHIBITION (*F*_2,31_ = 4.934, *p* = 0.014), which indicated that with the tDCS stimulation of the right IFG, an increase of RI was associated with an increase in fluency, *b* = 3.501, *t*_2.31_ = 2.880, *p* = 0.007, 95% CI = [1.021, 5.980], whereas no effect emerged with the left IFG stimulation condition, *b* = 4.597, *t*_2.31_ = 1.255, *p* = 0.219, 95% CI = [−2.874, 12.068].

Finally, a three-way interaction between tDCS CONDITION, MINDSET, and RESPONSE INHIBITION emerged, *F*_2,31_ = 6.032, *p* < 0.01. With the left IFG stimulation, no interaction between MINDSET and RESPONSE INHIBITION emerged (*p* = 0.425), in contrast, in the right IFG stimulation condition, analyses revealed an interaction between MINDSET and RESPONSE INHIBITION, *b* = −0.124, *t*_2.31_ = −3.378, *p* = 0.002, 95% CI = [−0.199, −0.049].

As shown in Figure 2B, in the right IFG stimulation condition, a simple slopes analysis showed the moderator role of participants’ mindset on RI over its association with fluency, *b* = −0.124, *t* = −2.662, *p* = 0.017. Precisely, after the right IFG stimulation, an increasing score of RI was associated with a decrease in fluency at high participants’ mindset scores (i.e., growth mindset), *b* = 1.649, *SE* = 0.590, *t* = −2.792, *p* = 0.013, but not at medium (i.e., neither fixed nor growth mindset), *b* = −0.552, *SE* = 0.365, *t* = −1.511, *p* = 0.150, and at low (i.e., fixed mindset) mindset scores, *b* = −0.544, *SE* = 0.508, *t* = 1.072, *p* = 0.299.

## Discussion

In this article, we examined the possibility of mindset being an explicatory factor for the interaction between RI and the neuromodulation induced by tDCS on DT. Interestingly, the mindset levels acted as moderators on stimulation conditions (i.e., the effectiveness of shifting activity from left to right IFG and vice versa using tDCS) and RI on the change of fluency and flexibility but not originality. The question arises: Why do mindsets moderate the change in fluency and flexibility but not the change in originality? One could argue that fluency and flexibility reflect quantitative measures and are thus conceptualized in the mindset frame as reflecting the mental operations related to ideation quantity. Such an interpretation is not feasible for originality (a qualitative aspect of creative ideation). It is also possible that this effect is an artifact of our method, which requires participants to produce alternative uses for everyday objects in a limited period. Originality requires time to emerge; consequently, only low levels of originality can be captured in the short duration of the experiments.

Intriguingly, growth mindsets had opposite moderator effects on the change of DT when high levels of RI were present in both stimulation conditions (which shows the effectiveness of shifting activity). These effects on DT were manifested in reduced fluency but enhanced flexibility. For the R + L− stimulation condition, a decrease in fluency was associated with high levels of d’ at high mindset scores (i.e., a growth mindset). On the contrary, high fluency scores were associated in this stimulation condition with a growth mindset when associated with low d’ levels. A different effect emerged in the R−L+ condition, especially on the flexibility index: an increase in flexibility was associated with high mindset scores (i.e., growth mindset) when associated with high d’ levels. For example, growth mindsets determined an enhanced level of d’, which is required for such processing and necessitates a temporary weakening of RI as a source of cognitive control. Therefore, the mindset modulated the effect of RI on the effectiveness of the activity shift between the left and right IFG on ideational fluency and flexibility.

These differential effects of the cortical activity shift on fluency and flexibility observed using the opposing stimulation condition could be explained using the *transient hypofrontality hypothesis.* This construct is one of the leading theoretical frameworks for clarifying differential bilateral frontal cortical activations (Dietrich, 2004). Given this hypothesis, we can argue that fluency and flexibility perform opposing flow experience functions, and accordingly, the activity shifts differ in these two indices for processing unconscious information and creative ideation.

What does it mean that activation of anodal right IFG is coupled with cathodal left IFG for fluency, while anodal left IFG is coupled with cathodal right IFG for flexibility? A possible explanation is that this is due to the right IFG’s association with language, which resonates with the nature of fluency (Marini et al., 2016; Gao et al., 2020). In contrast, the left IFG is associated with intention, self-regulation, and planning, functions involved in approach motivation and action-oriented processing (Petrides and Milner, 1982; Knight and Grabowecky, 1995; Tomarken and Keener, 1998; Kuhl, 2000), which aligns more with the mental operations involved in flexibility.

Regarding the empirical evidence on how mindset affects activity shift in IFG and creative ideation in the context of RI using tDCS, the jury is out, as the picture is mainly incomplete, particularly in creativity literature and using non-invasive brain stimulation techniques. These are still early times for investigating creative neurocognition through neuroscientific techniques. A more extensive and critical discussion about the utility and validity of these methods in delivering key insights about brain function will necessarily impact how we view the relevance of these findings for the field of neuroscience of creativity.

## Concluding remarks, limitations and future directions

**Figure 3:**
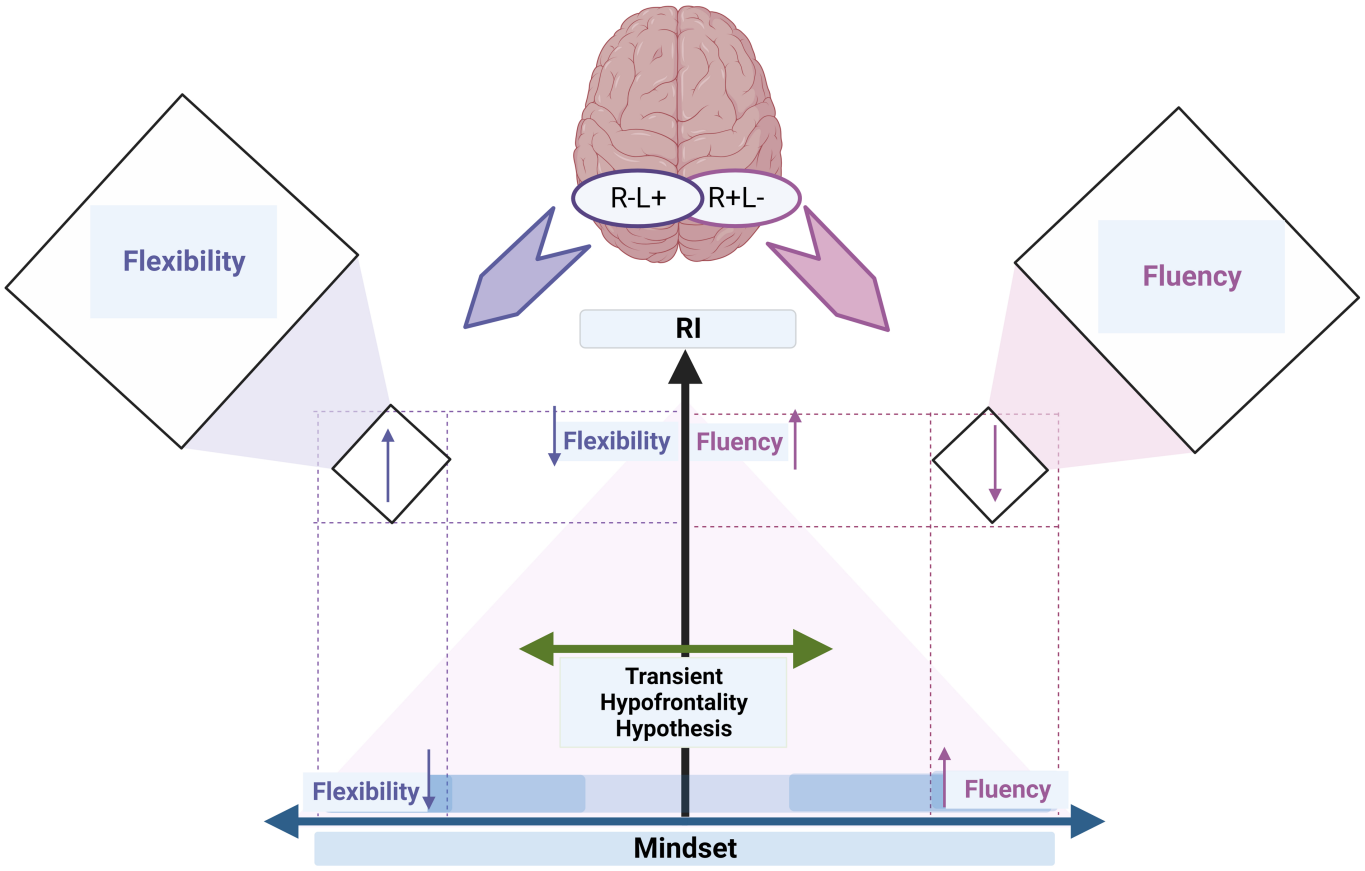
The summary graph illustrates how individual differences in creative ideation (fluency and flexibility) depend on cognitive control (i.e., RI) and cognitive status (i.e., mindset). The transient hypofrontality hypothesis (green left-right arrow) describes how stimulation can effectively shift cortical activity from left to right (R+L−) and vice versa (R−L+). The x-axis depicts mindset as a cognitive moderator on the activity shift of left/right and right/left IFG through the different levels of RI (y-axis). The y-axis refers to the increase in d’ of the RI level. The mindset axis refers to the increase in mindset scores from low (i.e., fixed mindset) to medium to high (i.e., growth mindset); the x-axis starts in the middle, directed by arrows, denoting an increase toward the left (flexibility) and right (fluency). The up and down arrows on the x-axis refer to the changes in fluency and flexibility when increasing mindset (i.e., growth mindset, x-axis) but low RI (y-axis). The up and down arrows on the y-axis refer to the changes in fluency and flexibility when increasing RI (i.e., d’, y-axis) but low mindset (i.e., fixed mindset, x-axis). The up and down arrows in the orthogonal boxes on the right and left sides refer to the changes in fluency and flexibility indices when high RI is associated with growth mindsets. The colors of the up and down arrows correspond to the colors of fluency (purple) and flexibility (blue).

Our report shows that mindset, specifically growth mindsets, act as a cognitive moderator on the activity shift of left/right and right/left IFG through the different levels of RI. This moderating role enhances flexibility and fluency, even if associated with high and low levels of RI, respectively (*cf.* Figure 3).

Brain non-invasive methods such as tDCS and its application to creative ideation can benefit from considering individual cognitive status (i.e., mindset) tendencies to provide valuable insights into ideation neural processes, mainly when RI is evaluated. Our findings provide the first exploration cues toward a research agenda in which mindset, RI, and creative ideation are closely intertwined using tDCS. Hence, a better understanding of the link between effective activity shifts in the frontal areas (i.e., IFG) and creative ideation in the context of RI will allow us to move to the second step and address open questions related to mental operations in creativity literature.

To date, our report presents a novel perspective on the moderator role of mindset (as cognitive status, particularly the growth mindset) on DT. This idea of cognitive status is rooted in the psychoanalytic tradition, where Harris (1953) suggested that creativity results from the ability to easily fluctuate between “primary-process” and “secondary-process” cognition, which was cited in Schnier and Kris (1953). On the one hand, the primary process is free-associative, analogical, and concrete. Generally, it occurs in states of distractibility like fantasy, reverie, and dreaming but also occasionally during abnormal states, as in some forms of psychosis. On the other hand, the secondary process reflects the abstract and logical thought processes grounded in conscious reality.

It is therefore relevant to highlight that our interpretations should be read in light of their limitations, particularly given that investigations of mindset in the creativity literature are still in a largely exploratory state. One of the challenges of implementing mindset in creativity research is that there are several definitions of mindset across different relational contexts (social psychology vs. educational psychology vs. cognitive psychology; Herz et al., 2020).

Although our findings are merely exploratory, they offer a direction to refine current conceptualizations in both creative ideation’s cognitive and neural perspectives. We considered the influence of individual differences and highlighted their signatures, through mindset, on shaping creative ideation in the context of RI. For these reasons, the nature of the context in which creative idea generation is called for the mental operations beyond it cannot be disregarded.

To assemble the puzzle regarding understanding neural correlates and cortical activation of creative ideation, neuroscience research agendas must consider the variety of neuroscientific methodologies (Abraham, 2013, 2018; Khalil et al., 2019, 2023a; Khalil and Moustafa, 2022; Khalil and Demarin, 2023). Some researchers established neuroscientific methods and imaging analysis techniques have yet to find wide use in creativity research, notably combined methods (e.g., simultaneous functional magnetic resonance imaging and electroencephalography; see Ullsperger, 2010). These fascinating techniques have been rarely implemented in the study of creativity. Moreover, the implementation of multivariate pattern analyses (Haxby et al., 2014), with a large sample of participants, and longitudinal approaches (Skup, 2010) could benefit creativity research, which is uncommon in the study of creativity (Schlegel et al., 2015). Estimating brain network structures using Bayesian approaches is another possibility. Durante and Dunson (2018) revealed that highly creative individuals often assemble inter-hemispheric connections. Consequently, the call for applying advances in neuroscientific methods could result in further progress in creativity research.

## Data availability statement

The raw data supporting the conclusions of this article will be made available by the authors, without undue reservation.

## Ethics statement

The studies involving humans were approved by the Ethical Committee at Constructor University Bremen. The studies were conducted following local legislation and institutional requirements. The participants provided their written informed consent to participate in this study.

## Author contributions

RK, AK, AAK, and BG conceptualized and conducted the research. SA and SM analyzed the data. RK, SA, and SM drafted the manuscript. All authors contributed to the article and approved the submitted version.

## Footnotes

^1^IC has generally been discussed as synonymous with inhibition, supporting a departure from functional fixedness by inhibiting typical or prepotent responses (Chrysikou, 2018, 2019; Palmiero et al., 2022).
