# Supplementary material for: Individual differences and creative ideation: neuromodulatory signatures of mindset and response inhibition
Source: Front Neurosci. 2023 Dec 6;17:1238165. doi: 10.3389/fnins.2023.1238165 (PMC10731982; doi:10.3389/fnins.2023.1238165)
Supplement: Supplementary file 1 [file Table_1.DOCX]

| Fixed effects | Estimated Coefficient | *SE* | *t* | *p* | CI | |
| --- | --- | --- | --- | --- | --- | --- |
|  |  |  |  |  | Low bound | Up bound |
| tDCS condition (R-L+ *vs.* R+L-) | -.741 | 2.247 | -.330 | .744 | -5.325 | 3.842 |
| Mindset✻R-L+ tDCS condition | .056 | .0326 | 1.703 | .099 | -.011 | .122 |
| Mindset ✻ R+L- tDCS condition | .046 | .0656 | .703 | .488 | -.088 | .180 |
| RI✻ R-L+ tDCS condition | .569 | .367 | 1.549 | .132 | -.180 | 1.318 |
| RI✻ R+L- condition | .249 | .4546 | .547 | .588 | -.678 | 1.176 |
| RI✻ Mindset ✻ R-L+ tDCS condition | -.016 | .009 | -1.734 | .093 | -.034 | .003 |
| RI✻ Mindset ✻ R+L- tDCS condition | -.011 | .016 | -.699 | .490 | -.043 | .021 |

Table SM1. Fixed effects of the GLM model on the change of originality
