# Supplementary material for: Individual differences and creative ideation: neuromodulatory signatures of mindset and response inhibition
Source: Front Neurosci. 2023 Dec 6;17:1238165. doi: 10.3389/fnins.2023.1238165 (PMC10731982; doi:10.3389/fnins.2023.1238165)
Supplement: Supplementary file 2 [file Data_Sheet_1.PDF]

Carol Dweck's Growth vs. Fixed Mindset Assessment

Place a check in the column that identifies the extent to which you agree or disagree with each statement.

	Strongly Agree	Agree	Disagree	Strongly Disagree
1. Your intelligence is something very basic about you that you can't change very much.				
2. No matter how much intelligence you have, you can always change it quite a bit.				
3. You can always substantially change how intelligent you are.				
4. You are a certain kind of person, and there is not much that can be done to really change that.				
5. You can always change basic things about the kind of person you are.				
6. Music talent can be learned by anyone.				
7. Only a few people will be truly good at sports – you have to be "born with it."				
8. Math is much easier to learn if you are male or maybe come from a culture that values math.				
9. The harder you work at something, the better you will be at it.				
10. No matter what kind of person you are, you can always change substantially.				
11. Trying new things is stressful for me and I avoid it.				
12. Some people are good and kind, some are not – it is not often that people change.				
13. I appreciate when parents, coaches, teachers give me feedback about my performance.				
14. I often get angry when I get feedback about my performance.				
15. All human beings without a brain injury or birth defect are capable of the same amount of learning.				
16. You can learn new things, but you can't really change how intelligent you are.				
17. You can do things differently, but the important part of who you are can't really be changed.				
18. Human beings are basically good, but sometimes make terrible decisions.				
19. An important reason why I do my schoolwork is that I like to learn new things.				
20. Truly smart people don't need to try hard.				

Circle the number in the box that matches each answer.

	Strongly Agree	Agree	Disagree	Strongly Disagree
1. Ability mindset – FIXED	0	1	2	3
2. Ability mindset – GROWTH	3	2	1	0
3. Ability mindset – GROWTH	3	2	1	0
4. Personality/character mindset – FIXED	0	1	2	3
5. Personality/character mindset – GROWTH	3	2	1	0
6. Ability mindset – GROWTH	3	2	1	0
7. Ability mindset – FIXED	0	1	2	3
8. Ability mindset – FIXED	0	1	2	3
9. Ability mindset – GROWTH	3	2	1	0
10. Personality/character mindset – GROWTH	3	2	1	0
11. Ability mindset – FIXED	0	1	2	3
12. Personality/character mindset – FIXED	0	1	2	3
13. Ability mindset – GROWTH	3	2	1	0
14. Ability mindset – FIXED	0	1	2	3
15. Ability mindset – GROWTH	3	2	1	0
16. Ability mindset – FIXED	0	1	2	3
17. Personality/character mindset – FIXED	0	1	2	3
18. Personality/character mindset – GROWTH	3	2	1	0
19. Ability mindset – GROWTH	3	2	1	0
20. Personality/character mindset – FIXED	0	1	2	3
**Total**				
**Grand Total**				

Strong Growth Mindset = 45 – 60 pts.

Growth Mindset with some Fixed ideas = 34 – 44 pts.

Fixed Mindset with some Growth ideas = 21 – 33 pts.

Strong Fixed Mindset = 0 – 20 pts.
